# Experience-dependent learning of behavioral laterality in the scale-eating cichlid *Perissodus microlepis* occurs during the early developmental stage

**DOI:** 10.1038/s41598-021-04588-8

**Published:** 2022-01-14

**Authors:** Yuichi Takeuchi, Yuna Higuchi, Koki Ikeya, Masataka Tagami, Yoichi Oda

**Affiliations:** 1 Department of Anatomy and Neuroscience, Faculty of Medicine, University of Toyama, Toyama, Japan; 2 World Freshwater Aquarium Aquatotto Gifu, Kakamigahara, Japan; 3 Graduate School of Science, Nagoya University, Nagoya, Japan

**Keywords:** Animal behaviour, Feeding behaviour, Learning and memory, Behavioural ecology

## Abstract

Behavioral laterality—typically represented by human handedness—is widely observed among animals. However, how laterality is acquired during development remains largely unknown. Here, we examined the effect of behavioral experience on the acquisition of lateralized predation at different developmental stages of the scale-eating cichlid fish *Perissodus microlepis*. Naïve juvenile fish without previous scale-eating experience showed motivated attacks on prey goldfish and an innate attack side preference. Following short-term predation experience, naïve juveniles learned a pronounced lateralized attack using their slightly skewed mouth morphology, and improved the velocity and amplitude of body flexion to succeed in foraging scales during dominant-side attack. Naïve young fish, however, did not improve the dynamics of flexion movement, but progressively developed attack side preference and speed to approach the prey through predation experience. Thus, the cichlid learns different aspects of predation behavior at different developmental stages. In contrast, naïve adults lost the inherent laterality, and they neither developed the lateralized motions nor increased their success rate of predation, indicating that they missed appropriate learning opportunities for scale-eating skills. Therefore, we conclude that behavioral laterality of the cichlid fish requires the integration of genetic basis and behavioral experiences during early developmental stages, immediately after they start scale-eating.

## Introduction

Although the human body is almost symmetrical, humans prefer to use one side of the body when performing sophisticated or powerful tasks as in the case of working with hands. "Behavioral laterality", i.e., preference for acting with one side of the body over the other, has been observed throughout the animal kingdom, as exemplified by tool use preference in chimpanzees and crows, paw preference in mice and toads, and preferential eye use in fish and cephalopods^1–11^. How does behavioral laterality arise? Handedness in human is likely attributable to the complex interactions between genetic and environmental factors^4^. Preferred hand-use has been observed since in utero^3^, and reports have suggested that it develops in childhood^12,13^. However, efforts to study the developmental process of human handedness have been hampered by difficulties in long-term observation, the inability to manipulate the body for experimental purposes, or due to social constraints (e.g., in some cases, a left-handed child is forced to write with his/her right hand)^14^. Animal-based studies have been limited to describing behaviors in adulthood, with the exception of the domestic chick in which pre- and postnatal influences were found on the brain and visual lateralization^15–17^. To date, the factors in animals that trigger hand preference and how lateralized behavior arises over an individual’s lifetime remain largely unexplored^18^.

In fish, behavioral laterality has been reported for predation and escape behaviors^19–26^, as well as response to conspecifics^27,28^. One of the most noticeable instances of behavioral laterality is exhibited by the “lefty” and “righty” scale-eating cichlid fish, *Perissodus microlepis*, in Lake Tanganyika^29,30^. The scale-eaters are abundantly and widely distributed in Lake Tanganyika and have become specialized at feeding predominantly on the scales of other fish through a mouth with skewed morphology^31,32^. Adults of this species attack prey fish from the preferred side to forage on the flank scales with their skewed mouths^33,34^. Lefty fish (approximately 50% of the population) have a larger lower jawbone on the left side, open the mouth slightly toward the right side, and attack the left side flank of prey fish, whereas the righty fish show the opposite behavior and morphology^35,36^. This left–right difference in mouth morphology is thought to be genetically determined^35,37,38^. Previous studies exploring the development of the lateralized predation behavior in this species have reported the following findings^39,40^: first, a large-scale analysis of the stomach contents and mouth morphology of *P. microlepis* in Lake Tanganyika revealed that juveniles begin scale-eating at a standard body length (SL) of 35–45 mm and the attack side preference gradually develops as the fish grow^39^. Large-sized adults with SL > 80 mm fed exclusively on scales and exhibited a well-established behavior of attacking from the dominant side of the predator. Second, naïve juveniles that were reared artificially with granular food (until 4 months after birth, reaching an approximate SL of 38–48 mm) and had no scale-eating experience, showed a weak preference of attacking prey fish from their dominant side during initial predatory attacks; however, with increasing scale-foraging experience, the fish improved their ability to remove scales efficiently by attacking from the dominant side^40^.

In this study, we determined whether the behavioral laterality in *P. microlepis* in Lake Tanganyika can be established only at a certain developmental stage or any stage, including adulthood, if the fish were given an opportunity to learn. Among songbird species, the zebra finch and white-crowned sparrow can reportedly learn their conspecific songs only during a short and specific period during early development^41,42^, whereas some starlings and nightingales can learn their songs until later stages^43,44^. Among animals in their native habitats, behavioral learning during development may be closely related to behavioral experiences in their life history^45,46^.

**Figure 1:**
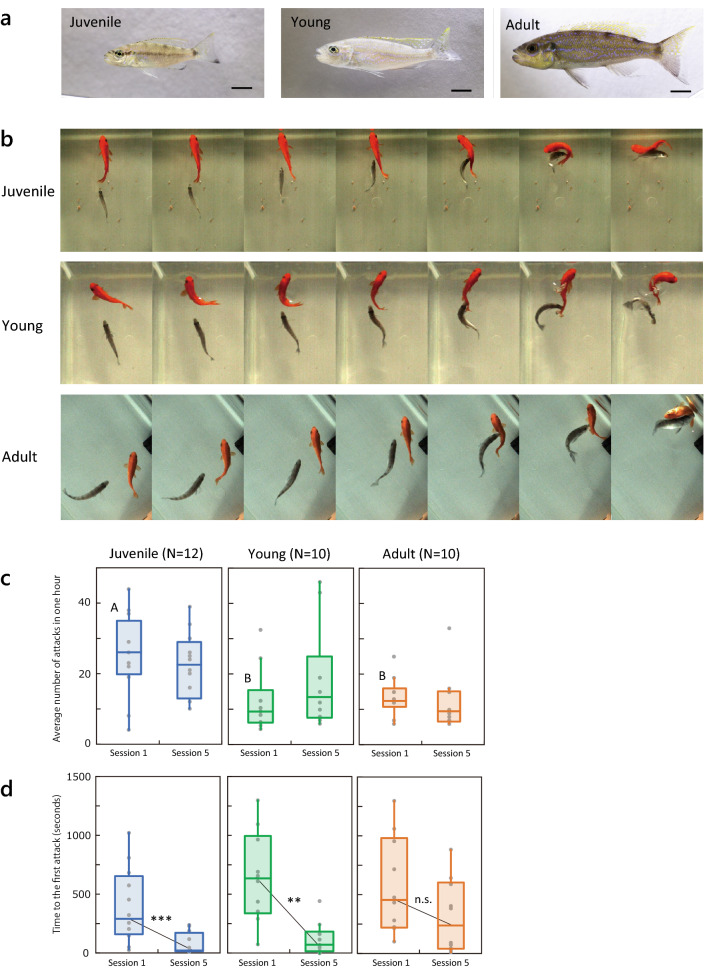
Scale-eating in naïve *Perissodus microlepis*. (**a**) Lateral view of juvenile (4 months old), young (8 months old), and adult (12 months old) cichlids reared in a tank without scale-eating experience. Bar: 1 cm. (**b**) Series of predation behaviors of three naïve lefty *P. microlepis* (gray) at different ages (Juvenile, Young, and Adult) in the first test session (Session 1). The predators are shown encountering prey goldfish (red) for the first time in their lifetime. (**c**) Number of attacks in 1 h during Sessions 1 and 5 at three stages of development (juvenile, N = 12; young, N = 10; adult, N = 10). “A” and “B” above the boxplots represent significant differences in pairwise comparisons (Tukey HSD test*, p* < 0.05). (**d**) Time to first attack by the predators after the introduction of prey fish into the tank. Wilcoxon signed-rank test was used to compare the time to initial attack in Sessions 1 and 5. **p < 0.01; ***p < 0.001. n.s., not significant.

To clarify the process of establishment of behavioral laterality, we compared the effect of scale-eating experience on acquiring behavioral laterality at three developmental stages (Fig. 1a): the “juvenile” stage (early developmental stage, SL ≤ 50 mm), when *P. microlepis* individuals start scale-eating; the “young” stage (51 mm ≤ SL ≤ 64 mm), with a mixed diet where they eat both plankton and scales; and the “adult” stage (SL ≥ 65 mm), with established scale-eating. The three developmental stages were determined from their SL, which corresponded to that of wild fish at three diet stages. The definition of the adult stage was based on the correlation between cichlid body size and weight of the sexual gland^39^. We used naïve *P. microlepis* that were fed only granular food until three developmental stages after birth (see also *Predation Experiment* in “Methods”): 4 months (juvenile), 8 months (young), and 12 months (adult). Predation experiments were conducted to determine: (1) whether naïve *P. microlepis* at the three developmental stages retain the motivation for scale-eating without any previous experience, (2) whether they develop scale-eating behavior with experience, (3) whether they learn to attack from the dominant side corresponding to their mouth morphology, and (4) which kinetics of predation movement (i.e., the velocity and amplitude of body flexion, and approaching speed for prey) they develop with predation experience.

## Results

*Perissodus microlepis* were hatched in our laboratory and reared in isolated home tanks with granular pet food until the predation test (Fig. 1a). The naïve predators at the three stages of development attacked the prey goldfish spontaneously and foraged their scales when both species were placed in the experimental aquarium (Fig. 1b). The naïve scale-eating predators attacked the goldfish prey from the beginning of Session 1 (i.e., at the first encounter in their lifetime), indicating that the scale-eaters had an innate preference to attack prey fish. However, attack frequency in the first session (Session 1) differed among developmental stages. The Kruskal–Wallis test detected significant differences between three stages (χ^2^ = 8.63, *p* = 0.013). The number of attacks during a 1 h session was significantly higher in juveniles (4 months old) than in either young (8 months) or adult (12 months) fish (Fig. 1c, Tukey HSD test: *p* < 0.05). After repeated sessions, juvenile and young fish initiated attacks on prey fish soon after they became aware of the prey. Time to initiate the first attack after introducing the prey fish into the test tank of *P. microlepis* was significantly shorter in Session 5 than in Session 1 in juveniles and young fish, but not in adults (Fig. 1d, Wilcoxon signed-rank test, Juvenile: S = − 39.0, *p* < 0.001, Young: S = − 25.5, *p* = 0.006, Adult: S = − 12.5, *p* = 0.232). This indicates that juvenile and young fish, but not adults, had higher motivation for predation with increasing experience.

**Figure 2:**
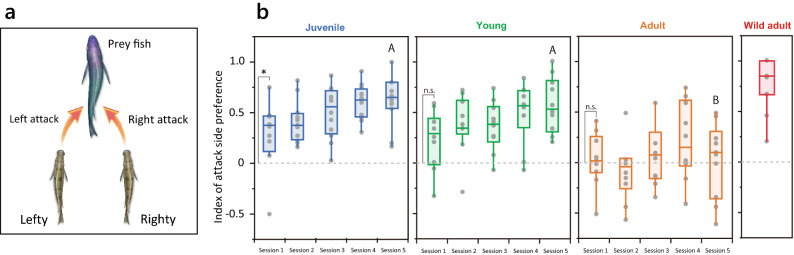
Attack side preference in *Perissodus microlepis*. (**a**) Diagram representing the link between attack side for scale-eating and mouth asymmetry of the predators. (**b**) Preference of attacks from the dominant side in Sessions 1–5. The initial attack side preference was tested in Session 1 using a one-sample Wilcoxon signed-rank test for deviation from chance (0.5). *p < 0.05. “A” and “B” represent significant differences in pairwise comparisons (Tukey HSD test, *p* < 0.001). The value for wild adult *P. microlepis* is shown on the extreme right (Takeuchi et al. 2012).

To test the laterality of predation behavior, we examined whether the attack side matched the dominant side of mouth morphology (Fig. 2a, also see “Methods”). In the first session (Session 1), the naïve juveniles attacked from a dominant direction more frequently than expected by chance (Fig. 2b Session 1, one-sample Wilcoxon signed-rank test: Z = 28.0, *p* = 0.026). In contrast, naïve young and adult fish did not express such preference in Session 1 (Young: Z = 19.0, *p* = 0.055, Adult: Z = 5.00, *p* = 0.742). This suggests that juveniles had an initial preference to attack from the dominant side that was gradually lost, and finally disappeared in adults that had grown up without scale-eating experience.

**Figure 3:**
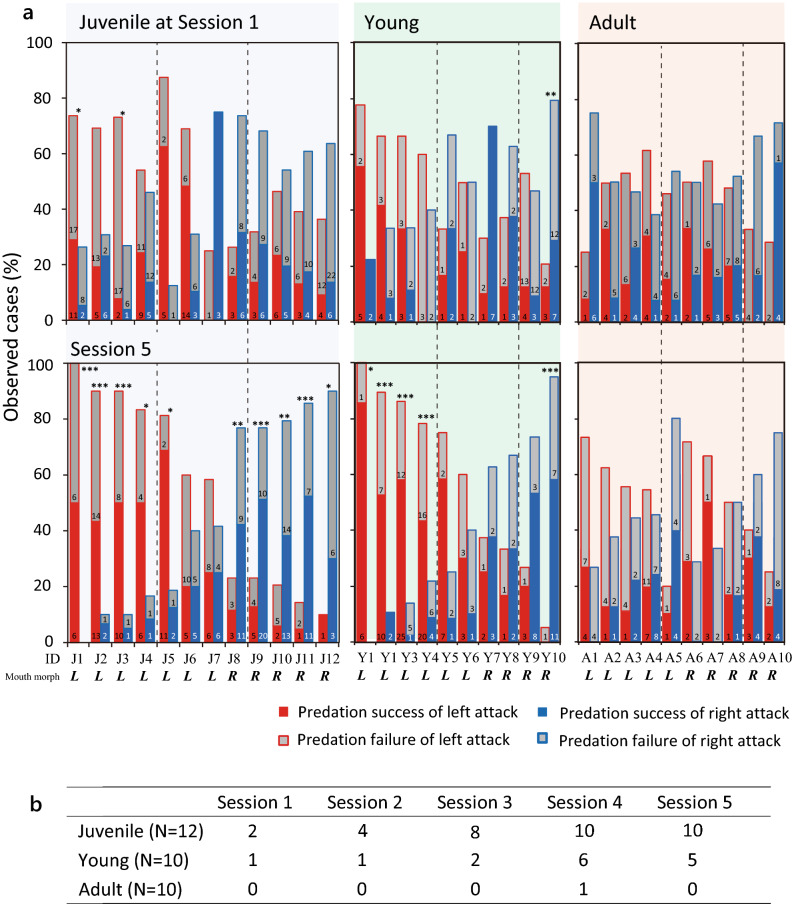
Strength of attack side preference at three developmental stages. (**a**) Proportion of left-side (red column) and right-side (blue column) attacks attempted by each predator (juveniles, J1–12; young, Y1–10; adults, A1–10). Red/blue and gray columns represent successful and failed predation attempts, respectively. Asymmetric mouth morphology—lefty (*L*) or righty (*R*)—is denoted at the bottom. *p*-values were calculated according to the binomial test. *p < 0.05; **p < 0.01; ***p < 0.001. (**b**) Number of individuals with significant bias in attack direction in Sessions 1–5 (binomial test, *p* < 0.05).

Next, to determine whether and how behavioral laterality developed with scale-eating experience during predation sessions, we measured an index of attack side preference (IAP). Juveniles and young fish progressively developed an attack side preference during five sessions for approximately 2 weeks (Fig. 2b; Spearman’s rank correlation, Juvenile, ρ = 0.457, *p* < 0.001; Young, ρ = 0.385, *p* = 0.006). We suppose that their attack side preference would have reached a level comparable to that of wild-caught adults (Fig. 2b, Wild adult) if they had undergone a few more test sessions. In contrast, naïve adults did not show any progress in attack side preference throughout the study period (ρ = 0.145, *p* = 0.316), and the value of adults in Session 5 was significantly lower than those of juveniles and young fish (Kruskal–Wallis test: χ^2^ = 14.39, *p* < 0.001; Tukey HSD test: *p* < 0.001). Additionally, we performed an individual-level analysis in each developmental stage. Among the juveniles, 2 of the 12 test subjects exhibited a significant attack side preference for the dominant side in Session 1 (binomial test, *p* < 0.05) (J1 and J3 in the left graph; Fig. 3a). In conjunction with population level analysis in Session 1, most individuals showed no significant lateral bias, while the group bias implies that the majority of attack direction is on the dominant side. The number of individuals exhibiting significant behavioral laterality increased over the five sessions, and the majority had acquired behavioral laterality by Session 5 (2, 4, 8, 10, and 10 of the 12 individuals tested in Sessions 1–5, respectively; Fig. 3a and b). In all cases, the attack side preference matched the dominant direction identified from the asymmetrical mouth morphology. Among naïve young fish, only one out of the ten subjects showed a side preference in Session 1 (Y10 in the middle graph; Fig. 3a). The number of individual young fish with significant behavioral laterality increased over the five sessions, but only half of the individuals showed a significant bias in Session 5 (1, 1, 2, 6, and 5 of 10 tested fish in Sessions 1–5, respectively). Thus, a significant bias in the attack direction was developed by only half of the young individuals but by more than 80% of the juveniles, indicating that learning efficiency may differ between the two age groups. In contrast to juveniles and young fish, almost none of the naïve adults showed a side preference during the sessions (0, 0, 0, 1, and 0 of 10 tested fish in Sessions 1–5, respectively): only one adult exhibited a significant attack side preference in Session 4, but this preference disappeared in Session 5. These results suggest that the ability to acquire behavioral laterality through experience is generally high at an age of 4 months (juvenile)—corresponding to the beginning of scale-eating behavior—but is gradually lost if the fish do not obtain scale-eating experience by the developmental stage of 8 months (young), and completely disappears by the age of 12 months (adult).

**Figure 4:**
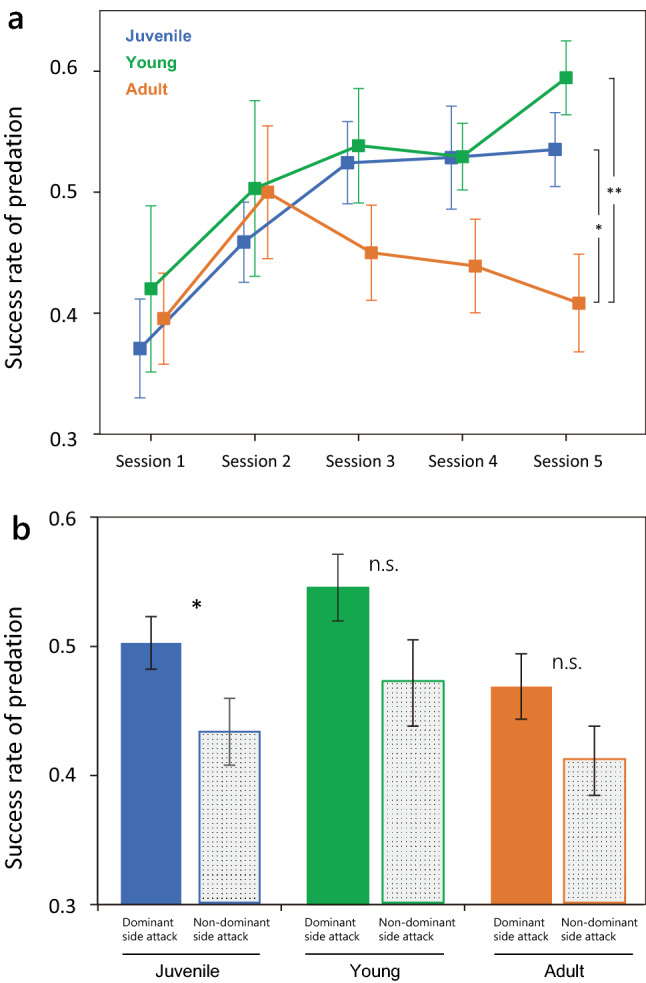
Successful predation during test sessions. (**a**) Changes in the success rate of predation from Session 1 to Session 5. Error bars indicate 95% confidence intervals. *p*-values for Session 5 are derived from generalized linear mixed model (GLMM) analysis. *p < 0.05; **p < 0.01. (**b**) Overall success rates of predation attempts from dominant and non-dominant sides (mean ± SE). *p*-values are from GLMM analysis. *p < 0.05.

Next, we investigated how learning lateralized movement contributes to predation success. Unexpectedly, we found that the success rate of predation increased with experience in young fish as well as in juveniles (Fig. 4a, GLMM analysis, Session 1 vs. Session 5, Juvenile: z = 4.38, *p* < 0.001; Young: z = 2.92, *p* = 0.004). In adult fish, by contrast, the success rate did not change significantly between Sessions 1 and 5 (z = 0.477, *p* = 0.634), although there was a transient increase in Session 2. In Session 5, the predation success rate of adults was significantly lower than those of juvenile and young fish (GLMM analysis, Adult vs. Juvenile: z = 2.30, *p* = 0.021; Adult vs. Young: z = 3.16, *p* = 0.002). Then, we determined whether the predation success was attributed to the learning of behavioral laterality by comparing the success rate between dominant-side attacks and non-dominant-side attacks. GLMM analysis showed that the success rate of juveniles was significantly higher in dominant-side attacks than in non-dominant-side attacks throughout all sessions (Fig. 4b, attack side: z =  − 2.30, *p* = 0.021, session: z = 4.62, *p* < 0.001). However, there was no significant difference in success rate between attack sides in young fish (attack side: z =  − 1.57, *p* = 0.117, session: z = 2.65, *p* = 0.008) and adults (attack side: z =  − 1.40, *p* = 0.163, session: z =  − 0.24, *p* = 0.808). These results indicate that juveniles learn to attack from the dominant side in order to effectively forage scales of the prey with their slightly skewed mouth, whereas young fish may have learned a different strategy to improve predation.

**Figure 5:**
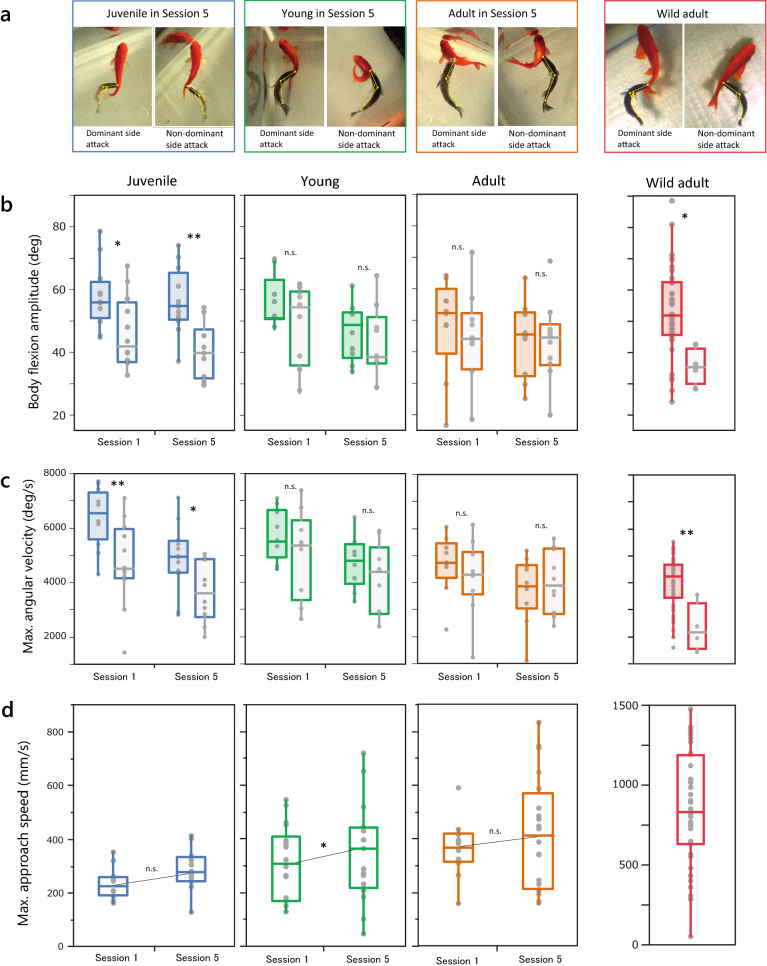
Kinematic differences between dominant- and non-dominant-side attacks at three developmental stages. (**a**) Representative pictures of dominant- (shaded) and non-dominant- (open) side attacks of three lefty predators at different stages of development in Session 5: juvenile, young, and adult. The rightmost images show predation by a wild adult (SL, 89.3 mm). Boxplots of the amplitude (**b**) and maximum angular velocity (**c**) of body flexion during attacks in Sessions 1 and 5 at different development stages (juvenile, N = 12; young, N = 10; adult, N = 10). (**d**) Maximum approach speed towards prey fish. The values of wild adults (N = 11) are shown on the extreme right. Wilcoxon rank-sum test was used for comparisons between dominant and non-dominant sides. *p < 0.05; **p < 0.01.

To further investigate the differences in predation between juvenile and young fish, we analyzed the kinetics of predation behavior in detail using a high-speed video camera. First, we focused on body flexion during the attack (Fig. 5a), which has been shown to differ between dominant-side and non-dominant-side attacks in wild-caught adults^36^. In juveniles, the amplitude of body flexion was significantly larger in the dominant-side attack than in the non-dominant-side attack in both Session 1 and Session 5 (Fig. 5b; Wilcoxon rank-sum test, Session 1, *p* = 0.019; Session 5, *p* = 0.003). The difference increased from Session 1 to 5. Similarly, the maximum angular velocity of juveniles was also higher in the dominant-side attack in both Session 1 and Session 5 (Fig. 5c; Session 1, *p* = 0.009; Session 5, *p* = 0.027). However, the dominant vs. non-dominant side difference was not observed in young and adult fish. There was no difference between the attack sides in body flexion amplitude and velocity in young and adult fish at either Session 1 or Session 5 (body flexion amplitude: Young, Session 1, *p* = 0.659; Session 5, *p* = 0.540; Adult, Session 1, *p* = 0.438; Session 5, *p* = 0.999; velocity: Young, Session 1, *p* = 0.427; Session 5, *p* = 0.348; Adult, Session 1, *p* = 0.270; Session 5, *p* = 0.791). These results indicate that left–right differences in body flexion for effective predation during scale-eating behavior are established through experience during the juvenile stage.

Furthermore, by observing whole movements of the predation behavior from the beginning of pursuit to the end of scale foraging, we found that young fish developed higher speeds when approaching the prey during the test sessions. The maximum approaching speed of young fish was significantly higher in Session 5 than in Session 1, although there were no lateral differences (Fig. 5d, GLMM, Young: dominated vs. non-dominant, t =  − 0.576, *p* = 0.566, Session 1 vs. Session 5, t = 2.131, *p* = 0.036). However, neither juvenile nor adult fish exhibited such changes in approach speed (Juvenile: dominant vs. non-dominant, t = 0.107, *p* = 0.915, Session 1 vs. Session 5, t = 1.957, *p* = 0.052; Adult: dominant vs. non-dominant, t =  − 0.860, *p* = 0.392, Session 1 vs. Session 5, t = 1.068, *p* = 0.288). Thus, the young fish learned to increase their approach speed regardless of their laterality, and improved their scale-eating efficiency, whereas neither juveniles nor adults acquired such experience-dependent acceleration when approaching prey. These results suggest that side differences in body flexion motions during predation and the attack side preference are initially acquired and developed through experience of scale-eating at the early developmental stage. In addition, the scale-eaters learned to accelerate the approach speed during the young stage. Wild adults probably improve both approaching speed and body flexion motions from the dominant side throughout the different stages of development. These skills are likely to contribute to successful foraging of scales of the prey. However, the inherent left–right differences and learning ability of the locomotive activity may be lost if the fish do not have scale-foraging experiences during the growing phases.

## Discussion

The present study aimed to clarify the role of scale-eating experience in the acquisition of behavioral laterality and the importance of developmental stage-dependent learning in the scale-eating cichlid *P. microlepis*, which exhibits lateralized predation behavior. The behavioral experiments showed that first, regardless of developmental stage, the scale-eating fish spontaneously attacked prey goldfish which they had never encountered before (Fig. 1b). Naïve juveniles (4 months old) in the initiation phase of scale-eating had an inherently strong motivation to forage prey scales and strengthen it through experience during the short-term test sessions. Naïve young fish (8 months old) were initially less motivated to attack but learned to become aggressive with experience. Adult fish (12 months old) without scale-eating experience tried to attack the prey fish, but their behavior did not develop with experience (Fig. 1c and d). Second, the naïve juvenile fish exhibited an innate preference for direction of attack and a more powerful attack motion on the dominant side (Figs. 2b, 5b, and c). They strengthened attack side preference and developed body flexion movement during the dominant-side attack and scale-eating experience, and their success rate of predation increased progressively (Figs. 3a and 4a). Third, the naïve young fish lost the initial lateralized preference, but gradually developed it through the scale-eating experience in some cases (Figs. 2b, 5b, and c). In addition, most of the young fish developed other predation skills that allowed them to quickly approach prey for successful predation (Fig. 5d). In contrast to the juvenile and young fish, the naïve adults raised without any scale-eating experience neither developed the lateralized motions nor showed a higher success rate of predation at the dominant side. Thus, although the scale-eating behavior could be initiated in all developmental stages from juveniles to adults, the development of scale-eating motivation and attack side preference depended on the developmental stage. The early life experiences may influence predation behavior in subsequent life stages. Notably, the juveniles and young fish improved their predation skills during the short periods of scale-eating experience (amounting up to 5 h in the laboratory), suggesting that during their brief experience at each developmental stage, *P. microlepis* learns to acquire the key motions of predation behavior. It should be emphasized that kinetics of the lateralized flexion movement during attack can be learned at the juvenile stage, immediately after the fish initiate scale-eating.

Previous studies on the life history of *P. microlepis* in Lake Tanganyika have shown that the larvae live in schools and feed on zooplankton, and that the juveniles (approximate SL, 45 mm) become isolated and gradually shift their feeding habits to the scales of prey fish^39,47^. The juveniles used in the present study showed dominant-side attacks more frequently than expected by chance at the first session (Session 1, Fig. 2b). When foraging flank scales of the prey, using a slightly skewed mouth, the preliminarily lateralized attack is advantageous to some extent and may accelerate the dietary transition to prey scales. Additionally, morphological asymmetry of the mouth may also develop as a result of scale-eating experience. Notably, wild individuals show more asymmetrical mouth morphology than artificially raised individuals (unpublished data). This is likely because the wild fish have more chances to attack prey than the fish grown in laboratory tanks. The consequential change in mouth morphology may further contribute to enhancing the dominant-side attack.

The juveniles and young fish learn different aspects of behavior with scale-eating experience: the earliest phase helps to learn lateralized attack behavior, while the middle developmental phase helps to gain speed for chasing. Remarkably, with scale-eating experience, the juvenile strengthened not only attack side preference but also developed body flexion movement during the dominant side attack. Notably, juvenile develop the naturally lateralized foraging behavior during the initiation phase of scale-eating; however, they gradually lose the inherent laterality if they are not provided opportunities of scale-eating during that period. In field, *P. microlepis* may learn different aspects of predation behavior at different developmental stages to maximize predation success and minimize the cost and risk associated with behavior expression (Figs. 5b, c, and d). Here, we provide a cautious conclusion regarding the difference in learning ability of attack direction between juvenile and young fish. Results of the binomial test suggested that the juveniles were better at learning attack direction compared to the young fish (Fig. 3b). However, since the results of binomial tests tend to be significant with a higher number of trials^48^, these results should be interpreted with caution. Therefore, the difference in learning ability for the lateralized attack between juvenile and young fish remains to be further clarified.

The specific period for learning during development is referred to as the “sensitive phase”^49^. It is well-known that some birds, such as ducks, cannot sing courtship songs as adults if they miss the opportunity to learn them through imprinting at a particular period^50–54^. Furthermore, the sensitive phase is believed to be associated with brain maturation^55,56^. Thus, different functions are learned at different stages of development. The visual responsiveness of the visual cortex neurons in kittens develops during different sensitive phases: binocular stereopsis develops during 3 to 15 weeks after birth, whereas orientation selectivity develops during 6 to 12 weeks^57–60^. Songbirds learn to sing conspecific songs during several phases of proficiency during development^61^. For example, zebra finches can produce sound elements of a model song by 65 days of age and can sing the precise order of these elements by 90 days of age^62^. Similarly, there is a sensitive phase in human language acquisition; to be able to speak a language effortlessly as a mother tongue, a child must live in the environment where that language is spoken and memorize the characteristics of the sounds used in that language by the age of 3–5^63,64^. Empirical evidence shows that second languages can be acquired with true fluency only within a sensitive phase from early infancy to puberty^65–67^. Taken together with the present findings on the scale-eating cichlid, these patterns indicate that a wide range of vertebrates learns different behavioral aspects at different developmental phases. The multiple sensitive phases per function may be related to the establishment of perceptual and cognitive abilities adapted to the growth environment of animals^46^. If the developmental stage-dependent learning of remarkably lateralized predation behavior in *P. microlepis* observed in the present study also occurs in the field, i.e., Lake Tanganyika, the cichlid may provide a new model system to study developmental learning in their native habitat.

The learning capabilities of teleost fish are comparable to those of mammals and birds in some cases, and the various taxonomic classes share a homologous neural network architecture^68–70^. Learning and memory play a crucial role in fish to establish prey recognition manipulation and ingestion efficiency^71,72^; however, stage-specific learning and its function have not been studied extensively. This study elucidates the currently neglected topic of the impact of ontogeny on behavioral laterality. In *P. microlepis*, behavioral laterality may be acquired specifically at an early developmental stage, triggered by scale-eating experiences, and represents an important advancement in our understanding of the establishment of ubiquitous behavioral laterality among vertebrates. *Perissodus microlepis* has a short lifespan, reaching sexual maturity in approximately 1 year. Previous studies have proposed the neuronal circuits underlying predation behavior, and genetic analysis of the lateral brain function is ongoing^36,73–75^. Genome sequences and gene expression data have been obtained from representatives of African cichlid fish—including those inhabiting Lake Tanganyika—such as species related to *P. microlepis*^76^. Overall, *P. microlepis* offers obvious advantages for investigating the developmental dynamics of predation behavior and their underlying neural and genetic bases. Given the abundance of available tools for investigating changes at the neurological and genetic levels in fish^11,77^, *P. microlepis* can pave the way to improve our understanding of the brain systems underlying phase-specific learning in predator–prey interactions.

## Methods

### Experimental animals

The scale-eating cichlid, *P. microlepis*, is an established model system for intraspecific dimorphism that is increasingly being studied for elucidating their behavior, ecology, genetics, and evolution^29,30^. The morphological asymmetry of the mouth opening occurs because one side of the joint is positioned frontward, ventrally and laterally, compared to the other side of the joint between the mandible and suspensorium^34^.

The scale-eaters used in the present study were obtained through breeding in our laboratory with help of the World Freshwater Aquarium Aquatotto Gifu (Japan). The broodstock were collected from Lake Tanganyika (Cameron Bay, Zambia; 8°29′S, 30°27′E) and transported to Japan by a fish dealer. As the offspring from one pair of wild fish were used in experiments, they were considered to have less genetic diversity. Hatching fish were incubated and housed individually in home tanks with air filters. The fish were maintained at 27 °C, pH 8.3, and a 12 h:12 h light: dark photoperiod with light provided by a 32 W fluorescent light. Naïve fish were fed daily with granulated food and small pellets only; thus, they never encountered prey fish before their predation experiments (Session 1–5). Food was not provided for 24 h preceding each experimental session to ensure a degree of hunger that would inspire maximum foraging performance.

### Predation experiment

Naïve scale-eaters at the ages of 4, 8, and 12 months after hatching were used for the predation experiments. The ages were determined from the SL of wild *P. microlepis* at three stages of diet that changed from plankton to scale-eating^47^: juvenile fish at the initiation of scale-eating (Fig. 1a; 44.6 ± 3.6 mm SL [mean ± SD], 4 months old, N = 12), young fish at the intermediate stage with a mixed diet (58.1 ± 1.9 mm SL, 8 months old, N = 10), and adult fish feeding chiefly on scales (68.0 ± 1.9 mm SL, 12 months old, N = 10).

The experimental aquarium (40 × 20 × 25 cm with a water depth of approximately 10 cm) was illuminated by two halogen lights (HVC-SL; Photron, San Diego, CA, USA) that were oriented diagonally to the aquarium. We covered the sides of the tank with translucent plastic so that the fish could not see the surroundings outside the tank. Additionally, the aquarium was surrounded by a blackout curtain so that the subject fish could not see the operator. Above the arena, a high-speed video camera (500 frames s^−1^, 1,024 × 1,024 pixels, NR4-S3; IDT Japan, Tokyo, Japan) was mounted to record the dorsal view of predation events. Simultaneously, the lateral view of predation events was monitored with a digital video camera (30 frames s^−1^, 1,920 × 1,080 pixels, HDR-XR550V; SONY, Tokyo, Japan) positioned 1 m lateral to the aquarium. By simultaneously recording the behavior of the fish from two directions, we were able to accurately capture the movement of both predator and prey even if they were close together.

Each subject fish was taken from its home tank and moved to the experimental aquarium. After 1 h of initial habituation, the predation session was started by introducing one prey goldfish (*Cyprinus carpio*; size 50–70 mm) into the tank. The scale-eater aggressively attacked the prey fish regardless of the prey’s body size. The predatory behaviors of scale-eaters during attacks on the prey goldfish appeared to be comparable to those of wild scale-eaters observed in Lake Tanganyika^36,78^.

For each predatory event, we recorded which side of the prey fish the predator attacked (left/right side) and the success or failure of the predation (hit/miss). A ‘hit’ was recorded if the mouth of the scale-eater contacted the body of the prey fish, and a ‘miss’ if not. When the predator contacted the prey fish, the prey fish curled the body to propel itself away quickly, so it was easy to determine whether contact had been made. At the end of the 1 h session, the scale-eater and the prey fish were gently captured and moved back to their respective home tanks. After the experiment, the scale-eaters were immediately given artificial food. All cichlids were observed to feed normally, indicating that fewer attacks made by some individuals were not due to lack of appetite.

The prey fish were changed for every trial. The scales lost by the goldfish through predation are regenerated in approximately 3 weeks^79^. To investigate the relationship between lateralized predation and experience, the predation experiments were performed in five sessions (Sessions 1–5) at intervals of 2–3 days. The gap between experiments was randomly assigned, regardless of the experimental groups. Between experiments, the fish were fed only pellets. Previous experimental data on wild adult fish^36^ were considered in the comparison of predation behaviors between developmental stages.

To assess the feeding motivation for predation, we measured the time from the introduction of the prey fish to the initial attack by the scale-eater. The degree of behavioral laterality during predation was calculated for each individual as the index of attack side preference (IAP), according to the following equation:

$${\text{IAP}} = \left( {{\text{Ad}} - {\text{An}}} \right)/\left( {{\text{Ad}} + {\text{An}}} \right),$$

where Ad is the number of attacks from the dominant side corresponding to each individual’s asymmetric mouth morphology, and An is the number of attacks from the non-dominant side during 1 h of predation.

The laterality morph of *P. microlepis* was determined based on the direction of the mouth opening^35,36^. After all the behavioral experiments, the cichlids were anesthetized in 0.01% MS-222 and each specimen’s mouth and craniofacial morphology were visually examined under a binocular microscope by two different researchers (Y. T. and Y. O.). A lefty fish was identified by the following three characteristics: the left lower jaw was larger than the right one, the left side of the head faced front, and the mouth opened rightward; a righty fish was identified by the opposite characteristics^37^. In general, all specimens used in the experiment were able to open their mouths wide in one direction or the other.

### Kinematics of scale-eating behavior

The recorded images of scale-eating behaviors were downloaded to a dedicated computer and digitized with a kinematics analysis software (Dipp-MotionV2D; Direct Co. Ltd., Tokyo, Japan). The swimming speed to approach the prey, the body flexion angle during attack, and the angular velocity were measured. The approaching speed was measured from the movement of snout. Body flexion angles were measured using three points on the midline of the body as shown in Takeuchi et al. (2012)^36^. These points were located at the snout, caudal peduncle, and center of mass^80,81^. The center of mass in *P. microlepis* was located in the anterior 38.3% of the body length^36^. Angular velocity was calculated by dividing the change in the flexion angle (observed in five sequential frames) by the time.

### Statistical analysis

Data were analyzed using JMP Pro 15 (SAS Institute Inc., Cary, NC, USA). All statistical tests were two-tailed, and alpha was set at 0.05. We compared the average number of attacks using Kruskal–Wallis test and Tukey–Kramer HSD test for each developmental stage. The difference in time to initial attack between Session 1 and Session 5 was examined using the Wilcoxon rank-sum test. The ecological relevance of laterality differs depending on whether it is found at the individual or population level^82^. At the individual level, the strength of attack side preference was evaluated in each individual scale-eater with binomial tests on the relative frequencies of dominant-side and non-dominant-side attacks. At the population level, Spearman’s rank correlation coefficient was calculated to test whether the IAP changed over time through repeated predation experiments in individual specimens. Further, to compare the strength of the behavioral laterality achieved after the test sessions at different developmental stages, we used Kruskal–Wallis test and Tukey–Kramer HSD test for the degree of IAP in Session 5. One-sample Wilcoxon signed-rank tests were used to establish how strongly each fish preferred to attack on the dominant side as opposed to choosing a side at random (50%). To examine how the predation experience contributed to predation success and which attack side was effective for success, we designed the generalized linear mixed model (GLMM) analysis with predation success (hit or miss) as the dependent variable and the following as independent variables: number of sessions (1–5) and attack side relative to mouth asymmetry (dominant side or non-dominant side) as the fixed effect and the individual as the random effect. Another GLMM analysis was performed to compare the predation success rate in Session 5 between adults and juveniles and between adults and young fish. Furthermore, the differences in predation success rate between Sessions 1 and 5 were analyzed using GLMM for three developmental stages. Wilcoxon rank-sum test was performed to compare the body flexion amplitude and the maximum angular velocity between dominant and non-dominant sides. To examine the effect of scale-eating experience on approaching motion, the maximum swimming speeds during prey approach were compared between Sessions 1 and 5 using GLMM analysis. These GLMM analyses were performed using R statistical software (version 3.6.2, R Statistical Computing, Vienna, Austria).

### Ethical note

The study was performed in compliance with all relevant ethical laws and guidelines in Japan and was supported and approved by the institutional review board in the Toyama University Committee on Animal Research (Approval # A2018MED-17)). Fish handling was performed under anesthesia with MS-222, and all efforts were made to minimize suffering. All procedures were conducted in accordance with the ARRIVE guidelines.

## Data Availability

The datasets analyzed in the current study are available from the corresponding author on reasonable request.
